# Lethal mutagenesis and evolutionary epidemiology

**DOI:** 10.1098/rstb.2010.0058

**Published:** 2010-06-27

**Authors:** Guillaume Martin, Sylvain Gandon

**Affiliations:** 1 Institut Des Sciences de l'Evolution de Montpellier, Université de Montpellier II—CNRS (UMR 5554), 34095 Montpellier Cedex 5, France; 2 Laboratoire de Génétique et Evolution des Maladies Infectieuses (UMR CNRS-IRD 2724 UR 165), 911 avenue Agropolis, 34394 Montpellier Cedex 5, France; 3 Centre d'Ecologie Fonctionnelle et Evolutive (UMR 5175), 1919 Route de Mende, 34293 Montpellier Cedex 5, France

**Keywords:** viral evolution, mutation load, fitness landscape

## Abstract

The lethal mutagenesis hypothesis states that within-host populations of pathogens can be driven to extinction when the load of deleterious mutations is artificially increased with a mutagen, and becomes too high for the population to be maintained. Although chemical mutagens have been shown to lead to important reductions in viral titres for a wide variety of RNA viruses, the theoretical underpinnings of this process are still not clearly established. A few recent models sought to describe lethal mutagenesis but they often relied on restrictive assumptions. We extend this earlier work in two novel directions. First, we derive the dynamics of the genetic load in a multivariate Gaussian fitness landscape akin to classical quantitative genetics models. This fitness landscape yields a continuous distribution of mutation effects on fitness, ranging from deleterious to beneficial (i.e. compensatory) mutations. We also include an additional class of lethal mutations. Second, we couple this evolutionary model with an epidemiological model accounting for the within-host dynamics of the pathogen. We derive the epidemiological and evolutionary equilibrium of the system. At this equilibrium, the density of the pathogen is expected to decrease linearly with the genomic mutation rate *U*. We also provide a simple expression for the critical mutation rate leading to extinction. Stochastic simulations show that these predictions are accurate for a broad range of parameter values. As they depend on a small set of measurable epidemiological and evolutionary parameters, we used available information on several viruses to make quantitative and testable predictions on critical mutation rates. In the light of this model, we discuss the feasibility of lethal mutagenesis as an efficient therapeutic strategy.

## 1. Introduction

Classical therapeutic strategies against viral infections rely on an arsenal of antiviral drugs that often have a narrow range of specificity (De Clercq 2002). An alternative and potentially generalist therapeutic strategy, called lethal mutagenesis, has been proposed in the last decade (Loeb *et al.* 1999) and theorized recently (Bull *et al.* 2007; Bull & Wilke 2008). Lethal mutagenesis is based on the statement that within-host viral populations can be driven to extinction when the load of deleterious mutations is artificially increased with a mutagen, and becomes too high for the population to be maintained. Because it targets a non-specific yet key feature of the pathogen biology (DNA or RNA replication) lethal mutagenesis could prove an efficient yet non-specific treatment strategy, also potentially less prone to the development of resistance (Freistadt *et al.* 2004).

It has been shown empirically that chemical mutagens can lead to important reductions of viral titres in cell cultures (reviewed in Anderson *et al.* 2004; Bull *et al.* 2007). The treatment of hepatitis C with the antiviral drug ribavirin is also considered to act (at least partly) through mutagenesis (Crotty *et al.* 2001). Theoretical predictions, however, have so far not allowed us to state the potential of lethal mutagenesis as an efficient therapeutic treatment. Bull *et al.* (2007) have clarified its general mechanism: when mutations have a deleterious average impact on fitness, the mean fitness of a population sets to a dynamic equilibrium between selection improving it, and mutation degrading it. This dynamic equilibrium is called mutation–selection balance (Haldane 1932; Burger 1998). If the equilibrium mean fitness is too low (i.e. in continuous time, when the mean Malthusian fitness 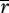 becomes negative), then the population size decreases deterministically to extinction.

A first limit to the understanding of lethal mutagenesis is that the evolutionary dynamics of virus populations have mostly been framed using unrealistic assumptions for the relationship between mutation and fitness. Indeed, most models applied to viruses make the simplifying assumption that fitness variation among alternative sequences is fully determined by the number of mutations they carry (or the ‘Hamming distance’ to a reference sequence, in sequence space). Two influential models (especially among virologists) fall into this category: Eigen's (1971) quasispecies model and all subsequent quasispecies theory (which often further limit to only two genotypic classes: a fit and an unfit), and Kimura & Maruyama's (1966) model, which served as the basis of Bull *et al.*'s (2007) theory of lethal mutagenesis. By construction, sequence space models do not allow for variation in fitness among genotypes with the same number of mutations, in contrast with all studies on the fitness effects of single mutations (Sanjuàn *et al.* 2004; Carrasco *et al.* 2007; Domingo-Calap *et al.* 2009). Besides, this simplification also impedes any form of compensatory mutations (that generate a fitter genotypic class). An experimental evolution study with bacteriophage T7 in the presence of a mutagen highlighted the importance of taking into account the occurrence of such beneficial mutations (Springman *et al.* 2009). Several recent studies attempt to take into account the effect of compensatory mutations with numerical simulations of mutation effects on RNA folding (Chen & Shakhnovich 2009; Stich *et al.* 2010), but with the drawback of equating fitness and protein stability (neglecting ecological aspects, gene regulation, etc.). Travelling wave models also provide a framework (analytical, this time) to study the effect of compensation (e.g. Manrubia *et al.* 2003; Rouzine *et al.* 2008). In these models mutations either reduce or increase fitness by a constant amount (±s). However, here too, both epistasis and fitness variation among mutations are ignored, and usually the occurrence of multiple mutants is neglected (i.e. low mutation rate approximation), which may be less valid for viruses.

In fact, an important class of models in evolutionary theory allows for both epistasis and variation in the fitness effects of mutations: phenotypic landscape models with an infinite pool of allelic effects, such as Fisher's geometric model (Fisher 1958). A main goal of this paper is thus to use this approach as an analytic framework that accounts for continuous fitness variation among mutants (including compensatory mutations), in a way compatible with available data. To do so, we borrow results from classical quantitative genetics theory. Following Kimura (1965) and Lande (1980), we consider that single mutations have a continuum of effects (normally distributed) on an arbitrary set of traits (continuum-of-allele model). These traits in turn determine fitness through stabilizing selection around an optimal trait value. Note that although this landscape has a single *phenotypic* optimum, it yields a very rugged *genetic* landscape with pervasive epistasis, following a pattern quantitatively consistent with available data on several microorganisms (Martin *et al.* 2007). Additionally, we also introduce a class of lethal mutations, absent from these models. In this context, the mutation load conforms to the Haldane–Muller principle (more precisely *L* = *U*) but only in the limit of low mutation rates (Burger 1998). However, when the mutation rate is high, simple predictions also exist for the distribution of standing genetic variation for the traits at mutation–selection balance (Lande 1980). As the assumption of high mutation rates seems reasonable in the presence of a mutagen, we propose to use this framework to describe the evolutionary dynamics of mean fitness under high mutation pressure.

A second limit to our understanding of lethal mutagenesis is that existing models for virus evolutionary dynamics have scarcely been set in an explicit demographic or epidemiological context. Usually, these models assume a constant population size, which is clearly incompatible with typical within-host dynamics. Only very few attempts have been made to couple evolution with the within-host dynamics (Boerlijst *et al.* 1996; Gerrish & Garcia-Lerma 2003; Bull *et al.* 2007; Bull & Wilke 2008) and they always relied on numerical simulations or on simplified description of the fitness effects of mutations (i.e. sequence space models described above). Therefore, another goal of this paper is to set the phenotypic landscape described above within an explicit demographic context. To do so, we use a simplified model that describes virus demography within a population of immunologically naive host cells (Anderson & May 1991; Nowak & May 2000).

In this paper, we present an analysis of the joint epidemiological and evolutionary dynamics of within-host infections. We show how epidemiology feeds back on the intensity of selection via the density of uninfected host cells (resources), and we show how in return evolution affects within-host dynamics via a reduction in mean fitness (mutation load). We derive the state of the system at epidemiological and evolutionary equilibrium, and show that the corresponding densities of infected cells follow a surprisingly simple linear relationship with mutation rate. We retrieve a simple closed form expression for the critical mutation rate at which within-host extinction should occur. Our predictions rely on several simplifying assumptions, but comparisons with individual-based stochastic simulations of within-host dynamics show the robustness of the analytical results. Following the approach of Bull *et al.* (2007), our results are expressed in terms of a few measurable quantities. The corresponding predicted values for the critical mutation rate in several viruses (based on available data on the distribution of fitness effects of mutations) suggest that an efficient reduction in viral titres could be achieved at realistic mutagenic levels. Finally, we discuss the implications of our results regarding the potential efficiency of lethal mutagenesis and the factors affecting it.

## 2. The model

### (a) Within-host dynamics and fitness landscape

We use a classical model of virus dynamics (e.g. Nowak & May 2000): we follow only the density of susceptible and infected cells, *S*(*t*)** and *I*(*t*)**, respectively, which in turn fully determine the dynamics of the viral titre *V*(*t*)**. This model relies on the fact that the viral titre is approximately proportional to *I*(*t*)**, with *V*(*t*)** = *I*(*t*)*k*_v_/*u*_v_, where *k*_v_ is the virus's burst size and *u*_v_ is the death rate of free virus particles outside host cells. This approximation is always valid at the epidemiological equilibrium, and is valid in the exponential phase (early infection) provided that the lifetime of an infected cell is much larger than the lifetime of a free virus particle (Nowak & May 2000). Transmission of the infection from cell to cell is then fully determined by a single parameter: the cell-to-cell transmission rate *β* = *β*_v_*k*_v_/*u*_v_, where *β*_v_ is the rate of entrance of free virus particles into new susceptible cells (Nowak & May 2000). This transmission rate is thus a synthetic measure of viral fitness over a large portion of its life cycle within the host. Note that we do not model coinfection here. This implies that both recombination and complementation between genotypes are not accounted for in the model. All these parameters and their definitions are summarized in the electronic supplementary material table.

We identify a given genotype by the vector **g** = [*g*_1_, … , *g*_*n*_] describing the genotypic value of this genotype at a set of *n* unknown viral traits that determine fitness and that may vary by pleiotropic mutation. The genotypic value **g** determines each genotype's cell-to-cell transmission rate *β*(**g**) via the following multivariate Gaussian function:2.1
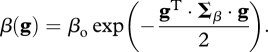



This corresponds to stabilizing selection around some optimum genotype set to **g** = 0. ***Σ***_*β*_ is an arbitrary positive semi-definite matrix describing the strength of stabilizing selection on all traits in **g**, and *β*_o_ = *β*(**0**) is the maximal transmission rate of the optimum genotype. The choice of **β**(**g**) in equation (2.1) guarantees that **β** is always positive (a negative **β** would have no biological meaning), and allows us to model mutation–selection balance in the classic context of stabilizing selection. Under the condition that the distance to the optimum 
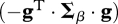
 remains small, however, the above fitness function is well approximated by the quadratic fitness function:2.2
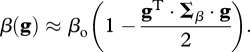

We now link this genotype-dependent transmission rate to fitness in order to define a fitness landscape. Denote by *I*(*t*, **g***)* the density of cells infected by genotype **g**, and *S*(*t*)** the density of susceptible cells. Denote by 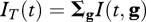 the total density of infected cells, and 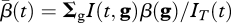 the mean transmission rate in the population. It can be shown (Gandon & Day 2009) that even when there is phenotypic variation among viruses, the dynamics of *S*(*t*)** and each genotype specific *I*(*t*, **g**) are given by the following differential equations:2.3
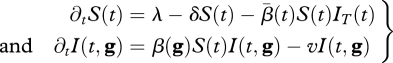

where *λ* and *δ* are the birth and death rates of susceptible cells and *ν* is the death rate of infected cells. The fact that all viruses are assumed to have the same death rate implies that fitness differences among viruses are only owing to differences in their transmission rates *β*(**g**).

Let us now focus on the key quantity linking evolution and demography: Malthusian fitness *r*(*t*, **g**) (for genotype **g** at time *t*). In our model, the Malthusian fitness of the viral genotype **g** is (using equations (2.3) and (2.2))2.4
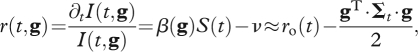

where 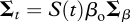 is the matrix of selective covariances and 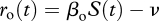 is the Malthusian fitness of the optimal genotype (**g** = 0) at time *t*. This form of stabilizing selection where Malthusian fitness (log fitness) is approximated by a quadratic function of genotypic values recalls the classical Gaussian multivariate fitness landscape used in quantitative genetics (Lande 1979). We will thus be able to use known results on the equilibrium distribution of **g** in this context. From now on, we will use the term fitness to denote Malthusian fitness.

Note, however, one important distinction with the classical fitness landscape. Although the transmission rate **β**(**g**) is constant for any genotype, the overall Malthusian fitness is not: it depends on the demography (through *S*(*t*)) generating a form of density-dependent selection. Increased availability of resources (uninfected cells) tends to (i) increase the maximal fitness attainable by the population (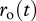) and (ii) increase the strength of selection between alternative genotypes (***Σ***_*t*_).

### (b) Mutation models

Mutations affect fitness in two ways. First, a portion *p*_*L*_ of mutations deemed ‘true lethals’ destroy the ability of the virus to be transmitted (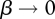). Second, a portion (1 − *p*_*L*_) of mutations affect fitness in a continuous manner by altering the viral phenotypic traits **g**. Hence, in contrast to true lethals, this second type of mutations stands a non-zero chance of surviving to the next generation and they can be compensated by a second mutation, if the latter moves the genotype back towards the optimum. The continuous effects of all mutations except true lethals are modelled following Kimura's (1965) continuum-of-allele-model: mutations create a random multivariate displacement in genotypic value **g** → **g** + ***ɛ***_**g**_, distributed as an unbiased multivariate Gaussian with arbitrary positive semi-definite covariance matrix **M**. As we are neglecting coinfection, we ignore the possibility of recombination and consider the viruses as asexuals, so that **M** describes the distribution of effects of mutations scattered over the genome. This Gaussian assumption for mutation effects on **g**, together with a quadratic Malthusian fitness function relating **g** to fitness (equation (2.4)), naturally accounts for variation in fitness among single mutants and for fitness epistasis (and therefore compensation), contrary to sequence space models. By contrast, true lethals cannot be modelled in the continuum-of-allele-model. The occurrence of true lethals is described by a simple unidirectional mutation process towards the lethal class (no compensatory mutations are allowed).

We considered two alternative models to describe the process of mutation, further detailed in the electronic supplementary material, appendix S1. First, we use a model (*Constant mutation model*) where mutations appear randomly as a Poisson process, evenly distributed over time and among genotypes. The number of mutations per genome per unit time follows a Poisson distribution with parameter *U*. Second, we use an alternative model (*Infection–dependent mutation model*) where mutation in any genotype **g** is conditional on the event of an infection of a new cell by this genotype. Indeed, one may argue that it is more realistic to assume that viruses can only mutate when they undergo an infectious cycle. When such an event occurs, the number of mutations produced by the infecting genotype follows a Poisson distribution with parameter **μ**, the mutation rate per infectious cycle. In this model, the mutation rate now depends on the probability of undergoing an infectious cycle, which varies among genotypes and over time (depending on cell densities). We show in the electronic supplementary material, appendix S1, that this model can be equated to the standard Poisson process (*Constant mutation model*), simply replacing *U* by a time-dependent effective mutation rate *U*_e_(*t*) = *μ*(*r̄*_*t*_ + 2*ν*). Therefore, under this mutation model, the effective mutation rate depends on the growth rate of the population and on the duration of the cell infection via the parameter *ν*. At demographic equilibrium, the mean growth rate of the population is 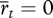 and the effective mutation rate is simply *U*_e_ = 2**μ*ν*. As for demographic parameters, all genetic parameters and their definition are summarized in the electronic supplementary material table.

### (c) Relating the landscape to empirical measures of effects of mutations on fitness

The *n*-dimensional phenotypic space of **g** described above corresponds to a set of unknown (and somewhat idealized) phenotypic traits that affect viral fitness. In order to parameterize the model in terms of measureable quantities, as in Martin & Lenormand (2006), we relate mutation effects on **g** to distributions of mutation effects on fitness components, that are amenable to experimental measurements. Viral fitness is typically estimated as a growth rate on a layer of susceptible cell cultures or within a naive host. Therefore, selection coefficients are typically estimated by the relative growth rate of a mutant in competition with its wild-type parent, the latter being usually well adapted to the study environment (genotype **g** ≈ 0). The mutant phenotype is thus given by **g**′ = **g** + ***ɛ***_**g**_ = ***ɛ***_**g**_ which is distributed as a multivariate Gaussian ***ɛ***_**g**_∼*N*(0, **M**). This selection coefficient *s*(*t*, **g**′) = *r*(*t*, ***ɛ***_**g**_) − 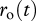 is a difference in Malthusian fitness and, in our model, depends on resource availability *S*(*t*) (see equation (2.4)). In order to avoid this time-dependence, it is usual to measure the relative growth during the exponential phase of the infection characterized when the availability of the resource is maximal. In our simple model, the maximal density of susceptible cells is *S* = *S*_max_ = *λ*/*δ*. Thus, when resources are not limiting so that *S*(*t*) ≈ *S*_max_ remains constant over a period of time, the selection coefficient remains roughly constant through time. For a mutation arising on an optimal parent genotype the selection coefficient is (from equations (2.3) and (2.4))2.5
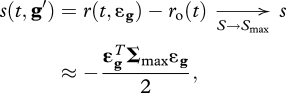

where ***Σ***_max_ = 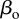 *S*_max_ ***Σ***_*β*_ is the matrix of selective covariances at the maximal host cell density. All along, note that *r*_o_ (without time variable) will denote the growth rate of the optimal genotype at maximal host cell density (*S*(*t*) = *S*_max_).

Defined in this way and under the additional assumptions on fitness landscape (i.e. quadratic fitness function) and on mutations (i.e. small Gaussian mutation effects on **g**) detailed above, the predicted effects of mutation on *s* are well approximated by a gamma distribution, in agreement with available distributions in viruses (Domingo-Calap *et al.* 2009). We use this relationship between landscape parameters and *s* to express our predictions in terms of the parameters of the distribution of *s* among single random mutants: its mean (in absolute value) *s̄* = |*E*(*s*)| ≥ 0 and its shape *α* = 1/CV(*s*)^2^, where CV(*s*) is the coefficient of variation of *s* among mutants. This is detailed in the electronic supplementary material, appendix S2.

We have defined above true lethals which cannot transmit to new cells. It is worth distinguishing this type of mutant from ‘apparent lethals’ that refer to viruses with a non-zero transmission rate but with negative growth. Indeed, the transmission rate of apparent lethals is so low that it cannot compensate for the death rate of infected cells, even at the highest cell density *r*(**g**) = *β*(**g**)*S*_max_ − *ν* < 0. Note that with the Gaussian function relating transmission to **g** (equation (2.1)) no mutation can lead to exactly *β* = 0 as is the case for true lethals. In a typical experiment, however, these two types of mutations would be undistinguishable, as none of these mutants would grow from a small inoculum. In contrast to true lethals, however, apparent lethals may be rescued and become viable in a context with a higher availability of resources (higher *S*_max_). This latter type of lethal may or may not form an important portion of lethal mutations observed empirically in viruses, but only true lethals are counted in the proportion *p*_*L*_. In the electronic supplementary material, appendix S3 we show how to estimate the proportion of apparent and true lethals from empirical distributions of fitness effects.

### (d) Stochastic simulations of within-host dynamics

We made various approximations in our derivations of the model presented above. In order to test the robustness of our analysis we used exact individual-based stochastic simulations, describing the fate of competing asexual virus genotypes in the demographic context described above (detailed in the electronic supplementary material, appendix S4). These simulations avoid several approximations that were made in the analytical derivations: they allow for demographic stochasticity (as opposed to the deterministic model presented above) and thus also encompass stochastic fluctuations in allele frequencies, i.e. genetic drift. They also allow for any strength of selection, whereas weak selection is assumed in our derivations (quadratic term on the right-hand side of equation (2.1)). Finally they allow checking of our analytical treatment of the *infection-dependent mutation model* (the electronic supplementary material, appendix S1, *U*_e_ = *μ*(*r̄*_*t*_ + 2*ν*)). These exact simulations thus allow a full test of the validity of our various approximations. All simulation codes were developed with the software R (R Development Core Team 2007) and are available upon request.

## 3. Results

### (a) Eco-evolutionary feedbacks and equilibrium

The strength of selection ***Σ***_*t*_ which determines the equilibrium distribution of **g** depends on the host cell density *S*(*t*) (see equation (2.1)). This host cell density itself sets the value of the mean transmission rate *β̄*(*t*) (as described in the electronic supplementary material, appendix S2) which in turn influences *S*(*t*) and *I*_*T*_(*t*). These feedbacks make it difficult to derive the time dynamics of the system, but the final equilibrium state can be derived. This state is characterized by two mutually dependent equilibria: (i) an evolutionary equilibrium for the distribution of **g** and the corresponding mean transmission rate β̄_*_, and (ii) a demographic equilibrium for cell densities S_*_ and *I*_T*_. This paired equilibrium, hereafter denoted eco-evolutionary equilibrium, is jointly solved in equation (A2.8) of the electronic supplementary material, appendix S2.

The mean transmission rate at equilibrium β̄_*_ and the equilibrium density of susceptible cells S_*_ jointly verify the evolutionary equilibrium (equation A2.7 in the electronic supplementary material, appendix S2) and the demographic equilibrium: S_*_β̄_*_ = *ν* (equation (2.3)). Solving this system yields expressions in terms of the basic mutational and demographic parameters. The mean transmission rate of the viral population is decreased relative to the optimal genotype by an amount:3.1
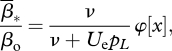

where 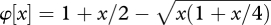 and 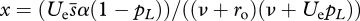 depends on the mutation rate *U*_e_, which depends on the mutation model: *U*_e_ = *U*, while *U*_e_ = 2 *μν* in the infection-dependent mutation model. The function *φ*[*x*] decreases from 1 in the absence of mutation (*x* = 0), to 0 when the effective rate of mutation becomes very large (*x* → ∞), so that the average transmission rate varies between 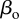 and 0 as the mutation rate increases. The corresponding equilibrium densities are given by the equilibria of the epidemiological model (from equation (2.3)). At eco-evolutionary equilibrium, the density of susceptible cells is S_*_ = *ν*/β̄_*_, and the density of infected cells is: *I*_T*_ = *λ*/*ν* − *δ*/β̄_*_ where β̄_*_ is given by equation (3.1).3.2
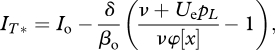

where *φ*[*x*] is defined as above, and 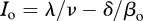 is the equilibrium density of infected cells in the optimal genotype. Recall that the corresponding virus titre in the free stage (blood, organ, etc.) is approximately proportional to this density.

A simple approximation to the above results can be found whenever the effective mutation rate and growth rate of the optimal genotype are large relative to the death rate of infected cells (*U*_e_, *r*_o_ ≫ *ν*): *U*_e_ ≫ *ν* is expected for a virus, at least when treated with a mutagen, and *r*_o_ ≫ *ν* is expected for a virus that shows acute infections in the absence of treatment. Factor *x* in equations (3.1) and (3.2) then becomes independent of *U*_e_ and the transmission rate then decreases proportionately to 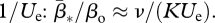 The constant3.3
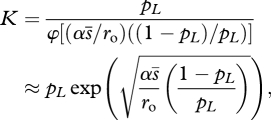

where function *φ*[.] is given in equation (3.1), depends only on the growth rate of the optimal genotype (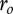) and on mutational parameters for both non-lethal (*α*, *s̄*) and lethal mutations (*p*_*L*_), all of which are experimentally amenable. From equation (3.3), the value of the factor *K* in the absence of ‘true’ lethal mutations (limit as *p*_*L*_ → 0), i.e. when non-lethal mutations are the main driving force of lethal mutagenesis is 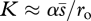. On the contrary, when true lethal mutations are the main driving force (*p*_*L*_ ≫ *s̄*), the factor becomes *K* ≈ *p*_*L*_. The equilibrium density of infected cells also simplifies in these conditions, the infected cell density then decreases approximately linearly with mutation rate, starting from its value in the optimal genotype:3.4
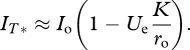



Overall, we see that in spite of the complexity of the system, the decrease in infected cell density and equilibrium virus titre is an approximately linear function of the effective mutation rate *U*_e_[Fig RSTB20100058F1].

**Figure 1. RSTB20100058F1:**
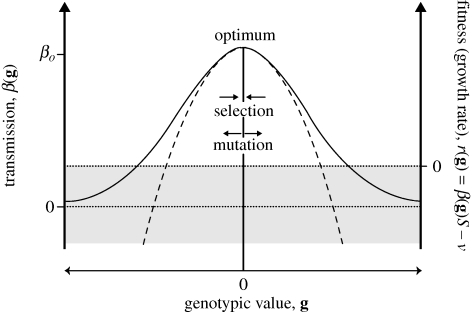
The fitness landscape. The mechanism generating a mutation load in the fitness landscape model is illustrated below in one dimension, in a case where a single trait **g** determines (Malthusian) fitness, *r*(**g**) = *β*(**g**)*S* − *ν*. Fitness depends on within-host transmission rate, *β*(**g**), which is assumed to be a Gaussian function (full line) which can be approximated by a quadratic function (dashed line) near the optimum (see equations (2.1) and (2.2)). Fitness depends also on the density of susceptible cells, *S*, and the death rate of infected cells, *ν*. In the grey area, fitness is negative. Mutations that fall in this grey area are thus called apparent lethals. In addition we allow a fraction *p*_*L*_ of mutation to be true lethals (not represented on this figure). Mutation produces phenotypic variance around the mean phenotype, which lies close to the optimum (**g** = 0), while selection reduces this variance. This sets an equilibrium distribution for **g** to which corresponds an equilibrium distribution for *r*(**g**). When *r*(**g**) < 0 the population decreases, which may ultimately lead to its extinction (i.e. lethal mutagenesis).

**Figure 2.**
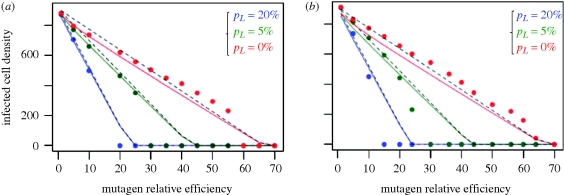
Within-host eco-evolutionary equilibrium. The effect of treatment by a mutagen on the final density of infected cells I*_T_ is represented with a proportion *p*_*L*_ (indicated on the graph) of true lethal mutations (with a zero transmission rate). After 50 time units at the natural mutation rate, the mutation rate was increased by a factor given on the *x*-axis (mutagen efficiency). The equilibrium state of the viral population is given for the two mutation models: (*a*) constant mutation or (*b*) infection dependent mutation. Each dot gives the average I*_T_ over *t* = 150 to 500 time units (at equilibrium with the mutagen induced mutation rate) in three replicate simulations. The distribution of fitness effects had shape *α* = 1.5 and mean *s̄* = 0.1. The epidemiological parameters were *λ* = 100, *ν* = 0.1, *δ* = 0.05 and *r*_o_ = 1 (corresponding to 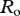 = 11). Solid lines show the theoretical value for *I*_T*_ (equation (3.2)) using either mutation rate (*a*) *U* or (*b*) *U*_e_ = 2 *μν* according to the mutation model. The decrease in infected cell density is approximately a linear function of mutation rate (or mutagen efficiency here), as illustrated by the accuracy of the linear approximation (equation (3.4), dashed lines) and simulations are indicated by filled black circles.

All these predictions are in agreement with stochastic simulations. Figure 2 shows that the equilibrium density of infected cells is well approximated by equation (3.2) and equally accurately by the linear approximation in equation (3.4) for any proportion of lethals and in the two alternative mutation models (setting the right value to *U*_e_). This agreement is further illustrated in the electronic supplementary material, figure S1 for two values of *s̄* in the absence of lethals.

### (b) Critical mutation rates

Next, in order to evaluate the feasibility of lethal mutagenesis, we sought to derive the critical mutation rate *U*_e_, above which the virus population will ultimately become extinct. Extinction is defined by the fact that the equilibrium total density of infected cells is zero: *I*_T*_ = 0. One can find this threshold value for *U*_e_ using either equation (3.2) (‘exact’) or its linear approximation (equation (3.4), both yielding the same threshold mutation rate:3.5
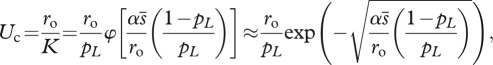

where *K* is given in equation (3.5) and function *φ*[*x*] has been defined in equation (3.1). We thus retrieve a criterion that only depends on the parameters describing the effects of mutations (*α*, *s̄*, *p*_*L*_), and on the growth rate of the optimal genotype (*r*_o_), with all these parameters being measured when the availability of susceptible cells is maximal (i.e. *S* = *S*_max_). When non-lethal mutations are the main driving force of lethal mutagenesis (limit as *p*_*L*_ → 0 in equation (3.5)) the critical mutation rate simplifies to 
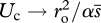
. On the contrary, when it is true lethal mutations that drive the process (*p*_*L*_ ≫ *s̄* in equation (3.5)), it simplifies to *U*_c_ → *r*_o_/*p*_*L*_. The criterion applies to the effective mutation rate *U*_c_. In the *constant mutation model*, it sets an extinction limit to the mutation rate per unit time: *U* ≤ *U*_c_. However in the *infection-dependent mutation model*, it sets a limit to the mutation rate per infection *μ* ≤ *μ*_c_ = *U*_c_/2*ν* where *U*_c_ is given by equation (3.5).

Figure 2*a*,*b* shows that, in stochastic simulations, extinction indeed occurs roughly at the limit set by equation (3.5) for both mutation models. Note that extinction tends to occur in fact at a slightly lower mutation rate than *U*_c_: equation (3.5) gives only a conservative (but still rather accurate) upper bound. Note also an important distinction between the two mutation models and theory. In neither of the two models does full extinction occur, only a very strong reduction in the density of infected cells. In fact, complete extinction would ultimately occur in all simulations, given sufficient simulation time, because of stochasticity. We now delve quickly into this issue of stochasticity with an example of an actual treatment.

### (c) Stochastic dynamics and treatment with a mutagen

**Figure 3.**
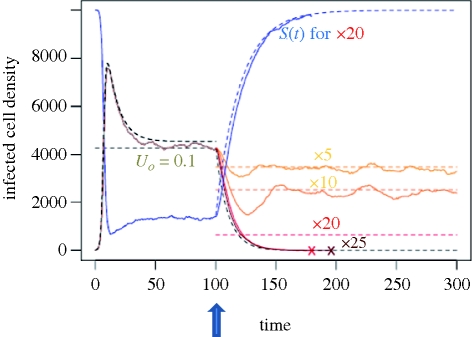
Effect of a mutagenic treatment on the course of an infection. The dynamics of the infected and susceptible host densities when a mutagen treatment is applied after the start and stabilization of an infection (curative treatment). The treatment consists in an increase in mutation rate relative to the natural rate (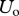 = 0.1, constant mutation model here). The dashed black curve gives the expected dynamics in the absence of mutation (one strain SIS) before the onset of treatment, at *t* = 100, indicated by the dark blue arrow. From that point each colour corresponds to different mutagen efficiencies (given on the graph). Plain lines give the simulated dynamics at each mutagen efficiency, and dashed lines show the corresponding predicted equilibrium (equation (3.2)): in the absence of extinction, simulated dynamics fluctuate around this prediction. The dashed red curve starting at the onset of the treatment, gives the expected fastest dynamics to extinction (exponential decrease at rate −*ν*). The dashed blue curve gives the corresponding expected fastest possible increase for *S*(*t*), with the plain blue curve giving the corresponding dynamics of *S*(*t*) in simulations with a mutagen of effect (×20). The coloured crosses give the time point of extinction in the treatment of the corresponding colour. Mutation effects parameters: shape *α* = 1.5, mean *s̄* = 0.1 with *p*_*L*_ = 20% of true lethals, predicted critical mutagen efficiency is *U*_c_/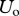 ≈ 23 from equation (3.5). Epidemiological parameters: same as figure 2 except *λ* = 500, and the infection was started at *I*(0) = 10 and *S*(0) = *S*_max_ = *λ*/*δ*.

In figure 3, we follow the dynamics, in a single stochastic simulation run, of infected and uninfected cells following a viral infection treated with a mutagen, after the onset of infection. The simulation starts with a small inoculum of the optimal genotype. This time dynamics also provides insights into the effect of stochasticity, as does the electronic supplementary figure S2 showing the time dynamics from the simulations used in figure 2.

In figure 3, before the onset of the treatment (indicated by the blue arrow), the density of infected cells follows closely the predicted epidemiological dynamics (grey curve) corresponding to the demographic parameters of the optimal genotype (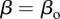). The equilibrium titre sets to a value slightly below the one expected for the optimal genotype, because the virus is assumed to have a non-zero natural mutation rate (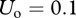 in our simulation) which already decreases the mean fitness of the viral population. Then, at time *t* = 100 the treatment with a mutagen starts; it consists in an increase in *U* by a given factor (or mutagen efficiency). After the start of the treatment the titre quickly drops to a new equilibrium value. The dashed line gives the corresponding predicted value of the equilibrium density of infected cells (equation (3.2)). Note that the extinction of the virus population occurs at slightly lower values of *U* than expected by our analytic model (*U*_c_ in equation (3.5)), as in figure 2. We believe the discrepancy between these simulations and our analytic predictions is owing to multiple consequences of stochasticity (finite population sizes).

First, the transient dynamics following the start of the treatment may lead to extinction before reaching the expected equilibrium density. Indeed, when the mutation rate is increased, the new evolutionary equilibrium is reached faster than the new corresponding demographic equilibrium for host cell densities. This will lead to an overshoot of the effect of mutagens on the mutation load which results in a decrease in the density of infected cells below the equilibrium density. When population sizes are small, this may lead to extinction very fast. This heuristic argument is illustrated in figure 3, for the curve ×20 mutagen efficiency (exponential decrease to extinction, black dashed curve).

Second, stochasticity will induce genetic drift and this alone is expected to affect the evolutionary dynamics. Indeed, our model only captures the deterministic part of the mutation load which results from the balance between mutation and selection. In finite populations, genetic drift will also reduce mean fitness relative to the optimal genotype.

Third, with finite populations, the population is in fact doomed to become extinct after sufficient time, whatever the mutation rate (as we could also confirm in our simulations, not shown). What varies, however, is the expected time to extinction. In this case, the concept of critical mutation rate needs to be redefined using a threshold value for the time taken to extinction. In this context, one may use the approximations for expected time to extinction from quasi-stationary distributions obtained by Nasell (2005).

The present deterministic theory therefore only provides an upper limit for the mutation rate above which extinction is fast. We expect that adding other stochastic effects (such as fluctuations in population sizes caused by external factors), would lead to even faster extinctions. Our theory thus provides a conservative prediction to evaluate the feasibility of lethal mutagenesis.

## 4. Discussion

### (a) Summary of the results

The present paper is an attempt to analyse the feasibility of lethal mutagenesis as a therapeutic strategy against viral infections, although the model could be readily extended to deal with other pathogens (Bull & Wilke 2008; Chen & Shakhnovich 2009). We combine an evolutionary model of mutations and stabilizing selection together with a demographic model of within-host dynamics (jointly modelling viral and host cell dynamics). In contrast to previous studies (Gerrish & Garcia-Lerma 2003; Bull *et al.* 2007; Bull & Wilke 2008), the present model assumes that single mutations have variable (gamma distributed) effects on Malthusian fitness (growth rate), in agreement with empirical data (Sanjuàn *et al.* 2004; Carrasco *et al.* 2007; Domingo-Calap *et al.* 2009). To do so, we use a fitness landscape model of stabilizing selection for an arbitrary (unknown) set of underlying traits. We then make use of the fact that, at the high mutation rate induced by mutagens, classical models of mutation–selection balance, based on a Gaussian distribution for the phenotype at equilibrium (Lande 1980), are accurate (Turelli 1984). These models thus provide a relevant and well-developed framework to study RNA virus fitness and phenotypes under the joint action of selection and mutation. For more realism, we also add a class of lethal mutations (not described in the former landscape models), in a way akin to sequence space models. In our model the population of virus genotypes shows variation for the number of mutations they carry (as in classical quasispecies theory), the fitness effect of each mutation (including beneficial ones), and even epistasis for fitness between mutations.

We used the classical model of virus within-host dynamics (Nowak & May 2000) to incorporate explicit within-host demography. Interestingly, taking this dynamics into account reveals two important consequences of demography on evolution. First, since uninfected host cells are the fuel of virus fitness, the intensity of selection depends on the availability of these cells. As the population of viruses decreases (e.g. following a treatment) the number of uninfected cells increases and the intensity of selection becomes stronger. This effect may help rescue a population on the verge of extinction. Second, demography may also affect the rate of appearance of new mutations. If mutations are conditional on the event of infecting a new host cell, the mutation rate necessarily depends on the birth and death rate of the population (*Infection-dependent mutation model*). A population with higher turn-over and higher growth rate generates a higher number of mutations per infected cells. This feedback of demography on evolution may be key to accurately predict within-host adaptation or extinction.

Our model is mainly used to focus on the joint demographic and evolutionary equilibrium of the within-host viral population. Although many biological factors have been introduced into the model, the resulting effect of mutation on virus titre and infected cell density proves surprisingly simple. At eco-evolutionary equilibrium, the total density of infected cells *I*_T*_ is an approximately linearly decreasing function of the effective mutation rate. If 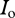 is the density of infected cells for an infection by the optimal genotype, then rearranging equations (3.4) and (3.5), yields simply4.1
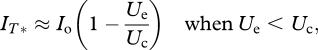

where *U*_e_ is the effective mutation rate depending on the mutation model and 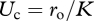 (equation (3.5)) is the critical mutation rate for extinction (where the constant *K* is given in equation (3.3)). This critical rate *U*_c_ only depends on the fitness effects of non-lethal mutations (*α*, *s̄*), and proportion of lethal mutations (*p*_*L*_), and on the maximal growth rate of the optimal genotype (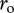). When the effective mutation rate is high enough (*U*_e_ > *U*_c_, equation (3.5)), the equilibrium cell density becomes so small that extinction is effectively certain within a short time. With finite populations, full extinction at *U* > *U*_c_ may not always happen instantaneously, but a very sharp decrease in population density is observed, at *U* values slightly below our theoretical *U*_c_, owing to stochastic effects (figures 2 and 3).

These results were confirmed, for both infection-dependent mutation or constant mutation models, by individual-based simulations that accounted for the stochasticity that was neglected in the model (figures 2 and 3). The mean transmission rate decreases proportionately to 1/*U*_e_ which was also confirmed by simulations (not shown).

### (b) Factors affecting the efficacy of the treatment

Our results, summarized in equations (4.1) and (3.5), yield qualitative insights into what aspects of the virus biology make it less or more prone to extinction by lethal mutagenesis. The efficacy of a mutagen in controlling an infection is fully determined by *U*_c_ (equation (3.5)): any factor decreasing *U*_c_ will facilitate the treatment, by implying a faster reduction of the virus titre at a given mutation rate *U*_e_ > *U*_c_ (equation (4.1)), and by allowing its extinction at lower mutagen efficacy. These factors are mutational effects parameters (*α*, *s̄*, *p*_*L*_) and a single demographic parameter, the growth rate of the optimal genotype *r*_o_ .

We illustrate in the electronic supplementary figure S3 how the parameters of mutation effects on fitness affect *U*_c_ and the treatment efficacy. First, not surprisingly, a virus with a lower proportion *p*_*L*_ of true lethal mutations is less prone to lethal mutagenesis. Lethal mutations have a strong impact on *U*_c_ (see figure 2 and the electronic supplementary material, figure S3), which means that a ‘biased’ mutagen that would also increase the frequency of lethal mutations would be much more efficient. Second, lethal mutagenesis may occur even in the absence of lethal mutations (*p*_*L*_ → 0, in equation (3.5)), at a critical mutation rate 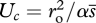, i.e. more likely so for a virus with a larger *s̄* or a larger *α*. This latter effect contrasts with previous models of lethal mutagenesis, based on sequence space models and where the mutation load was independent of mutation effects on fitness (Bull *et al.* 2007). It also concurs with results from the recent simulation study using protein stability as a proxy for fitness (Chen & Shakhnovich 2009). In this paper, fitness was fully determined by protein stability, which de facto creates a phenotypic landscape, where, as in our model, compensatory mutation takes place. Consistently, Chen & Shakhnovich also observed that mutation effects parameters affected the mutagen efficacy. The discrepancy with previous models based on sequence space is probably caused by the presence, in phenotype space landscapes, of compensatory mutations, epistasis between mutations, and variation in fitness among single mutants, that affect the mutation load and its demographic impact.

The only remaining parameter that influences the efficacy of the treatment (equation (3.5)) is the growth rate *r*_o_ of the infection (measured for the optimal genotype during early exponential phase). Not surprisingly, all else being equal, a virus with higher growth rate will be more difficult to cure, and this parameter alone summarizes the impact of demographic factors on the efficacy of lethal mutagenesis. In practical terms this means that the efficacy of lethal mutagenesis could be increased if another drug was used to reduce *r*_o_ (e.g. by killing specifically infected cells and/or by reducing the replication rate of the virus). Finally, note that when mutation only occurs during new infectious cycles, the death rate of infected cells (*ν*) also affects the efficacy of the mutagen treatment, in the sense that the critical mutation rate *per infectious cycle*, is given by *μ*_c_ = *U*_c_/2*ν* where *U*_c_ is given by equation (3.5). Therefore, an infection inducing higher cell mortality (larger *ν*) should be more sensitive to mutation, because it has a higher turn-over rate per unit time. However, the efficiency of the mutagen (relative to natural mutation rate) that is required for a given decrease in viral titre remains independent of *ν* for both mutation models (as *U*_c_/*U* = *μ*_c_/*μ*).

### (c) Feasibility of lethal mutagenesis

Beyond qualitative predictions, our results allow a discussion of the potential of lethal mutagenesis based on quantitative arguments, because they are expressed as a function of measurable quantities. This also makes them potentially testable. First, our predictions depend on classical epidemiological parameters of within-host dynamics for the optimal genotype (*r*_o_ and possibly *ν*). Second, they depend on the parameters describing the distribution of fitness of single random mutations, measured when the availability of susceptible cells is maximal (*α*, *s̄*, *p*_*L*_). Based on the empirical estimates available for the latter parameters in five virus species, we computed (equation (3.5)) the predicted critical mutation rate for extinction (*U*_c_ in table 1). This threshold is fairly small and surprisingly constant across species (of the order of 15 mutations per viral genome replication), in part because empirical estimates suggest a high and fairly consistent frequency of lethal mutations across virus species. Overall, such critical mutation rates seem reachable with mutagens that would increase mutation rates by one to two orders of magnitude. Furthermore, even when extinction is not achieved, our results suggest that increasing the mutation rate may still be an efficient treatment, as the relative decrease in virus titre at a given *U* is (1 − *U*/*U*_c_) (equation (4.1), figure 2). Overall, these results point to an encouraging potential for lethal mutagenesis to efficiently eliminate, or at least reduce, viral infections.

**Table 1.** Predicting the critical mutation rate of several viruses. Unshaded rows give empirical estimates from distributions of single mutants: total observed proportion of non-viable mutants (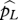), the mean of *s*_*ν*_ among viable mutants (*s̄*_*ν*_) and its variance *V*(*s*_*ν*_). Note that *s*_*ν*_ is a scaled measure of selection coefficient 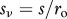 among viable mutants. Shaded rows correspond to theoretical predictions derived from these estimates, shape of the distribution among viable mutants (*α*_*ν*_), proportion of ‘apparent lethals’ (p*_L_, see the electronic supplementary material, appendix S3), proportion of true lethals (*p*_*L*_), critical mutation rate for extinction, (*U*_c_, equation (3.5)), as a function of the growth rate of the optimal genotype *r*_o_ (see the electronic supplementary material, appendix S3). Generation time (duration of an infectious cycle) is given in exponential growth, where 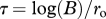, where *B* is the virus burst size. Units: g^−1^, per generation (inferred from generation time estimates); d^−1^, per day; h^−1^, per hour.

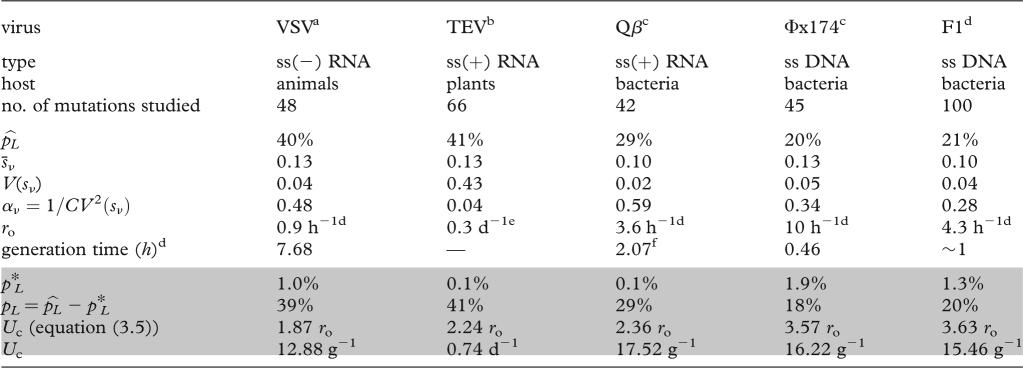

^a^Sanjuàn *et al.* (2004).

^b^Carrasco *et al.* (2007).

^c^Domingo-Calap *et al.* (2009).

^d^R. Sanjuan (2010), personal communication and this issue.

^e^S. Elena (2010), personal communication.

^f^Mean based on estimates of burst size *B* in other leviviruses like Q*β* (MS2 & R17) from De Paepe & Taddei (2006).

### (d) Evolution of resistance

As in any therapeutic strategies, lethal mutagenesis may lead to the evolution of resistance in pathogens (as discussed in Manrubia *et al.* in press). *In vitro* studies revealed two types of resistance mechanisms against chemical mutagens. First, resistance may occur through increased fidelity of the replication (i.e. lowering *U*_e_). For example, Pfeiffer & Kirkegaard (2003) describe the *in vitro* evolution of poliovirus resistance against ribavirin, a nucleotide analogue which induces mutations by allowing base mismatches. This resistance is caused by a single amino acid change in the viral polymerase that increases the fidelity of RNA synthesis and thus limits the mutagenic effects of the drug. Second, our results suggest that resistance could also occur via a decrease in the average fitness effects of mutations (i.e. lower *s̄*). This type of selection for lower *s̄* has been reported in VSV (Sanjuan *et al.* 2007). In the presence of the mutagen 5-fluorouracyl, a robust variant of VSV outcompeted the wild type strain, in the presence of a chemical mutagen, whereas the wild-type was the fittest in the absence of mutagen. The characterization of this robust variant revealed a lower effect of mutations on fitness but not a lower rate of mutation (Sanjuan *et al.* 2007). Overall, resistance to chemical mutagens is likely to occur and limit the feasibility of lethal mutagenesis.

The use of mutagens in combination with classical drugs may be a way to increase the efficacy of therapeutic treatments, and reduce the development of resistance (Gerrish & Garcia-Lerma 2003; Perales *et al.* 2007). We have seen here how reducing *r*_o_ (by means of a classical drug) would facilitate the treatment by a mutagen. However, the interplay between the effects of multiple drugs is still not well understood, and the evolution of resistance in this context even less. This clearly deserves both theoretical and experimental investigations.

### (e) Concluding remarks and perspectives

We hope that the theoretical framework proposed here may help model virus adaptation in a context that includes sufficient complexity to be reasonably realistic. An important feature of the present approach is that the predictions are testable because they are expressed in terms of a few quantities that can all be measured experimentally (table 1).

We believe, however, that the predictive potential of the present theory of lethal mutagenesis could be improved in several directions. First, as pointed out above, our simulations revealed a potential impact of stochasticity in the dynamics of the pathogens. Second, recombination and complementation have been neglected in our analysis. This is likely to affect our predictions on equilibrium densities of infected cells. These two factors, however, require coinfection and thus a relatively high density of infected cells. This implies that their effect on the critical mutation rate (i.e. close to extinction when the density of infected cells is very low) could be weak. Third, in a more realistic context, the mutagen would be applied in a heterogeneous host environment, with some areas where the drug is not delivered. These untreated areas could form an important source of mutation-free genotypes able to rescue the virus population from extinction. This scenario has been recently modelled in a simplified model with only lethal mutations (Steinmeyer & Wilke 2009), it would be interesting to extend this work to include non-lethal mutations and viral adaptation to the different environments. More generally, predictive models of viral evolution in a heterogeneous host environment might be needed if we are to provide quantitatively useful models for medical applications. Fourth, as discussed above, we believe the potential synergism between chemical mutagens and more classical therapeutic drugs (Gerrish & Garcia-Lerma 2003; Perales *et al.* 2007) derves more work.
